# Two new species and a new combination in *Aculithus* Liu & Li, 2022 (Araneae: Phrurolithidae) from South China

**DOI:** 10.3897/BDJ.13.e153747

**Published:** 2025-04-08

**Authors:** Zimin Jiang, Zhongjing Wang, Xiaoting Lyu, Jun Yang, Keke Liu

**Affiliations:** 1 Key Laboratory of Jiangxi Province for Biological Invasion and Biosecurity, Jinggangshan University, Ji'an, China Key Laboratory of Jiangxi Province for Biological Invasion and Biosecurity, Jinggangshan University Ji'an China; 2 Wuyuan Forest Birds National Nature Reserve, Shangrao, China Wuyuan Forest Birds National Nature Reserve Shangrao China

**Keywords:** Jiangxi Province, hotspot, taxonomy, diversity

## Abstract

**Background:**

Jiangxi Province has gradually become a hotspot for research on the family Phrurolithidae Banks, 1892, with 64 new species discovered in the past five years. Notably, over half of the currently known *Aculithus* species (5 species) were recorded in this region. However, studies have primarily focused on southern Jiangxi, while northern Jiangxi and remote mountainous areas remain under-surveyed. Many unknown Phrurolithidae species are still likely to be discovered within the Province.

**New information:**

Two new species, *Aculithuslanguan* Jiang & Liu, **sp. nov.** and *A.ruijin* Jiang & Liu, **sp. nov.**, are described from Jiangxi Province, China. Morphological illustrations, SEM pictures, photos of live specimens and a distribution map are given. Additionally, a new combination, *A.taoyuan* (Fu, Chen & Zhang, 2016) **comb. nov.**, is proposed. The total number of the known species of *Aculithus* from China is raised to 12, including the three species treated in the present paper.

## Introduction

The spider family Phrurolithidae Banks,1892 comprises 26 genera and 414 valid extant species globally, with *Otacilia* Thorell, 1897 being the most diverse genus containing over 140 valid species (World Spider Catalog 2025). However, the taxonomic delimitation of *Otacilia* has long been contentious, primarily because the male of the type species, *O.armatissima* Thorell, 1897, has never been recorded, making male palp characteristics unavailable for comparison (Thorell 1897). In the past five years, there have been some significant changes and many species have been re-assigned to newly-established genera, effectively reducing the heterogeneity of *Otacilia* and offering fresh insights into the systematics of Phrurolithidae (Jiang et al. 2025). Still, the taxonomic status of many *Otacilia* species remains to be reviewed.

At present, 222 valid species from 18 genera of this family are recorded from China. The number of recorded species in China has doubled over the past five years, largely attributed to increased Chinese arachnologists' attention and support (World Spider Catalog 2025). Most species are concentrated in southern provinces (Jiang et al. 2025). Jiangxi Province is a focal area due to intensive recent surveys, with 65 species known to date (Jiang et al. 2025). However, significant geographical biases persist: while southern Jiangxi is relatively well-studied, northern Jiangxi and remote mountainous regions remain under-explored. These areas, characterised by complex habitats and minimal anthropogenic disturbance (Liu et al. 2022), likely harbour numerous undescribed taxa. Systematic sampling and integrative studies are urgently needed to uncover this hidden diversity.

The genus *Aculithus* Liu & Li, 2022 was established with the proposal of three new species and four new combinations (Liu et al. 2022). Subsequently, Mu and Zhang (2023) described a new species, *A.brevispinus* Mu & Zhang, 2023 and proposed a new combination, *A.pseudofabiformis* (Liang, Li, Yin, Li & Xu, 2021), during their revision of the genus *Otacilia* (Mu and Zhang 2023). No further related studies have been conducted since then. Recently, during investigations of Phrurolithidae spiders in Jiangxi Province, two undescribed *Aculithus* species were discovered. The aim of the present paper is to provide the description of these two new species and propose a new combination from the genus *Otacilia*.

## Materials and methods

Specimens were examined using a SZ6100 stereomicroscope. Both male and female copulatory organs were dissected and examined in 80% ethanol using an Olympus CX43 compound microscope with a KUY NICE CCD camera. Epigynes were cleared with pancreatin solution (Álvarez-Padilla and Hormiga 2007). For SEM photographs, the specimens were kept under natural dry conditions, coated with gold with a small ion-sputtering apparatus ETD-2000 and photographed with a Zeiss EVO LS15 scanning electron microscope. Types are deposited in the Animal Specimen Museum, College of Life Science, Jinggangshan University (ASM-JGSU).

The measurements were taken using a stereomicroscope (AxioVision SE64 Rel. 4.8.3) and are given in millimetres. The body lengths of all specimens exclude the chelicerae and spinnerets. Terminology of the male and female genitalia follows Liu et al. (2022).

Leg measurements are given as total length (femur, patella, tibia, metatarsus, tarsus). The abbreviations used in the figures and text are as follows: ALE − anterior lateral eye, AME − anterior median eye, Bu − bursa, CD − copulatory duct, CO − copulatory opening, CT − connecting tube, DTA − dorsal tibial apophysis, Em − embolus, FA − femoral apophysis, FD − fertilisation duct, GA − glandular appendage, MOA − median ocular area, MS − median septum, PLE − posterior lateral eye, PME − posterior median eye, rTA − retrolateral tegular apophysis, RTA − retrolateral tibial apophysis, SD − sperm duct, Spe − spermathecae.

## Taxon treatments

### Aculithus languan

Jiang & Liu sp. nov.

D8C1A8E3-45BB-552D-BB13-79D6866B2FC0

488AD87C-B499-4C82-9932-99B94399EEB8

#### Materials

- **Type status:** Holotype. **Occurrence:** catalogNumber: Phu-178; recordedBy: Liu Ke-Ke; individualCount: 1; sex: female; lifeStage: adult; occurrenceID: E3509C28-A8A8-52B3-8CF6-B2A373512088; **Taxon:** scientificName: *Aculithuslanguan* **sp. nov.**; **Location:** country: China; stateProvince: Jiangxi; locality: Shangrao City, Wuyuan County, Wuyuan Forest Birds National Nature Reserve, Dazhangshan, Wolong Valley; verbatimElevation: 533 m; verbatimCoordinates: 29°31'28.84"N, 117°44'39.07"E; georeferenceProtocol: GPS; **Event:** samplingProtocol: sieving; eventDate: 3/2/2025
- **Type status:** Paratype. **Occurrence:** catalogNumber: Phu-178; recordedBy: Liu Ke-Ke; individualCount: 1; sex: female; lifeStage: adult; occurrenceID: 208348DE-58CD-5180-8C27-980666884DD4; **Taxon:** scientificName: *Aculithuslanguan* **sp. nov.**; **Location:** country: China; stateProvince: Jiangxi; locality: Shangrao City, Wuyuan County, Wuyuan Forest Birds National Nature Reserve, Dazhangshan, Wolong Valley; verbatimElevation: 533 m; verbatimCoordinates: 29°31'28.84"N, 117°44'39.07"E; georeferenceProtocol: GPS; **Event:** samplingProtocol: sieving; eventDate: 3/2/2025

#### Description

**Figure 1.**
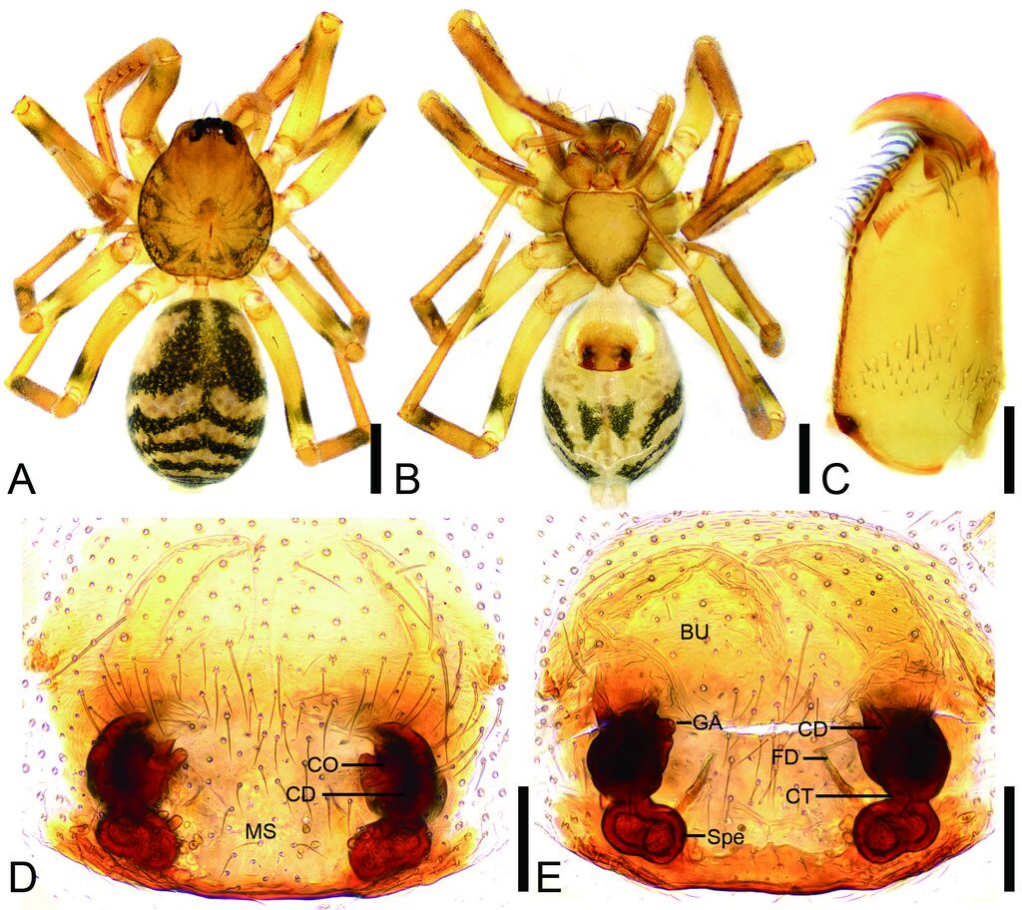
*Aculithuslanguan* **sp. nov.**, female (holotype). **A** Habitus, dorsal view; **B** Same, ventral view; **C** Left chelicera, ventro-retrolateral view; **D** Epigyne, ventral view; **E** Vulva, dorsal view. Abbreviations: Bu − bursa, CD − copulatory duct, CO − copulatory opening, CT − connecting tube, FD − fertilisation ducts, GA − glandular appendage, MS − median septum, Spe − spermathecae. Scale bars: 0.5 mm (A, B), 0.1 mm (C−E).

**Figure 2.**
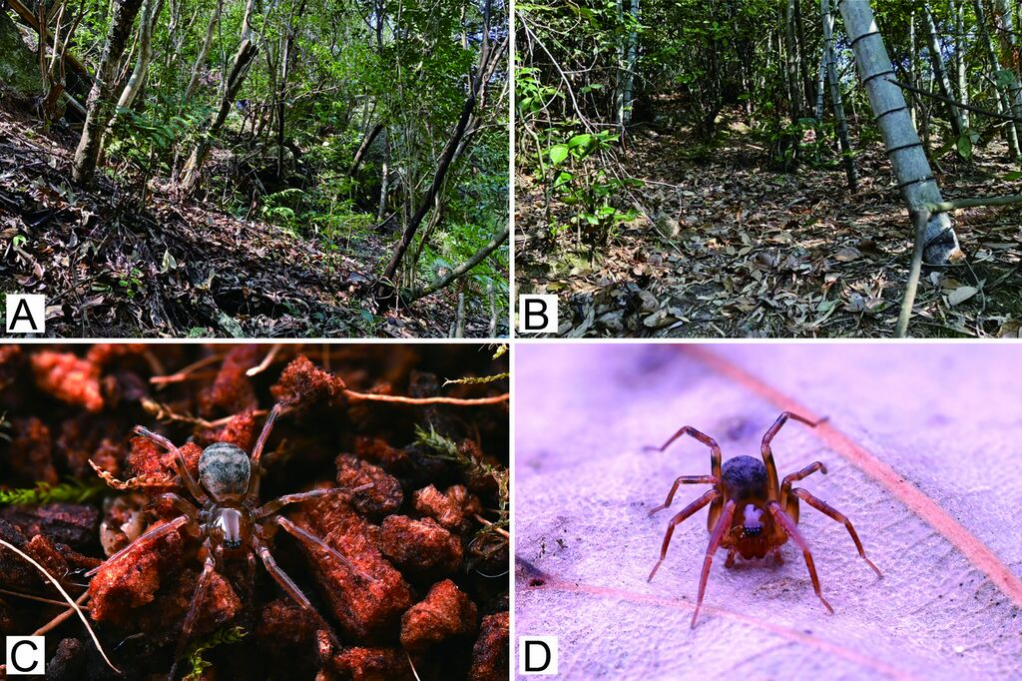
Habitat and live specimen of *Aculithuslanguan* **sp. nov.**. **A**–**B** Habitat; **C**–**D** Female.

**Female** (Holotype). Habitus as in Fig. 1A and B and Fig. 2C and D. Total length 2.81, carapace 1.23 long, 1.06 wide.

Eye sizes and interdistances (Fig. 1A): AME 0.05, ALE 0.08, PME 0.04, PLE 0.07; AME−AME 0.03, AME−ALE 0.02, PME−PME 0.07, PME−PLE 0.08, AME−PME 0.08, AME−PLE 0.14, ALE−ALE 0.14, PLE−PLE 0.26, ALE−PLE 0.05. MOA 0.15 long, frontal width 0.11, posterior width 0.14. Chelicerae (Fig. 1C) with three promarginal (proximal largest, distal smallest) and seven retromarginal teeth (the distal one largest, gradually decreasing in size, compact). Sternum (Fig. 1B), longer than wide, posteriorly triangular, relatively blunt. Leg measurements: I 3.77 (1.02, 0.47, 1.1, 0.79, 0.39); II 3.47 (0.93, 0.42, 0.82, 0.84, 0.46); III 3.19 (0.9, 0.37, 0.59, 0.81, 0.52); IV 4.32 (1.13, 0.42, 0.92, 1.23, 0.62). Left leg spination (Figs. 1A and B): femora I d11, p111, II d1, III d1, IV d1; tibiae I v222222, II v222221; metatarsi I v2221, II v2221. Pedicel 0.14 long. Abdomen (Fig. 1A and B) 1.44 long, 1 wide.

Colouration (Fig. 1A and B). Carapace yellow, with radial, irregular dark stripes mediolaterally and arc-shaped dark stripes around margin. Fovea distinct, brown. Chelicerae, endites and labium yellow. Sternum yellow, margins with dark brown mottled spots. Legs yellow, femora I−IV distally each with black annulations; tibia II distally with blackish-brown mottled markings, tibiae III−IV distally with blackish-brown annulations. Abdomen yellowish, anteriorly with a pair of large C-shaped markings, covering more than half of the anterior region; posteriorly with five dark brown V-shaped stripes extending towards the abdomen and converging towards the spinnerets; venter posteromedially has a pair of dark brown triangular spots.

Epigyne (Fig. 1D and E and Fig. 5A). Epigynal plate weakly sclerotised. Copulatory openings (CO) small, oval, shorter than the length of copulatory ducts (CD), located anterolaterally, widely separated by median septum (MS). Copulatory ducts slightly longer and thicker than spermathecae (Spe), anteriorly with pair of translucent bursae (Bu) covering nearly half of the epigyne. Glandular appendages (GA) slightly thicker than fertilisation ducts (FD), near base of bursae, located on inner side of copulatory ducts. Connecting tubes (CT) extremely short and narrower than spermathecae, extending posteriorly to connect with spermathecae. Spermathecae peanut-shaped, directed medially. Fertilisation ducts longer than spermathecae, directed anteriorly.

#### Diagnosis

The females of the new species are similar to *A.taoyuan* (Fu, Chen & Zhang, 2016) **comb. nov.** in having the large, widely-spaced copulatory openings, the copulatory ducts broader and longer than the spermathecae and the extremely short connecting tubes and the spermathecae directed medially (see Fu et al. (2016): 283−284, figs. 9G−H and 10D−E), but can be separated from it by copulatory openings directed laterally (Fig. 1D and E) (vs. directed anteriorly), the indistinct median septum (vs. distinct median septum) (cf. Fig. 1D and Fu et al. (2016): 283, fig. 9G), the peanut-shaped spermathecae (vs. globular spermathecae) and the fertilisation ducts longer than spermathecae (vs. shorter than spermathecae) (cf. Fig. 1E and Fu et al. (2016): 283, fig. 9H).

#### Etymology

The specific epithet refers to the Chinese name of *Garrulaxcourtoisi* (Ménégaux, 1923), 'lan guan zao mei', a critically endangered bird species from the Wuyuan National Nature Reserve for Forest Birds; noun in apposition.

#### Distribution

Known only from the type locality in Jiangxi Province, China (Fig. 7).

#### Biology

It was collected from leaf litter in areas of broad-leaved forests and bamboo broadleaf mixed forests in hilly areas (Fig. 2A and B).

### Aculithus ruijin

Jiang & Liu sp. nov.

D31361FB-56B3-51B4-8576-ADD395F61D18

1E0F6A4A-CF55-456A-B469-343D1E8F51E9

#### Materials

- **Type status:** Holotype. **Occurrence:** catalogNumber: Phu-173; recordedBy: Liu Ke-Ke; individualCount: 1; sex: male; lifeStage: adult; occurrenceID: 6F4B419E-A9D5-56AD-9D6F-859D589D7CD2; **Taxon:** scientificName: *Aculithusruijin* **sp. nov.**; **Location:** country: China; stateProvince: Jiangxi; locality: Ganzhou City, Ruijin County Level City, Jiubao Town, Tongbo Mountain; verbatimElevation: 834 m; verbatimCoordinates: 26°01'26.79"N，115°50'19.91"E; georeferenceProtocol: GPS; **Event:** samplingProtocol: sieving; eventDate: 2/8/2025
- **Type status:** Paratype. **Occurrence:** catalogNumber: Phu-173; recordedBy: Liu Ke-Ke; individualCount: 2; sex: male; lifeStage: adult; occurrenceID: 606461E8-84CB-5679-AF73-DC26779B76B4; **Taxon:** scientificName: *Aculithusruijin* **sp. nov.**; **Location:** country: China; stateProvince: Jiangxi; locality: Ganzhou City, Ruijin County Level City, Jiubao Town, Tongbo Mountain; verbatimElevation: 834 m; verbatimCoordinates: 26°01'26.79"N，115°50'19.91"E; georeferenceProtocol: GPS; **Event:** samplingProtocol: sieving; eventDate: 2/8/2025
- **Type status:** Paratype. **Occurrence:** catalogNumber: Phu-173; recordedBy: Liu Ke-Ke; individualCount: 6; sex: female; lifeStage: adult; occurrenceID: 7EB22543-B24B-57E8-9D58-16FA7479006E; **Taxon:** scientificName: *Aculithusruijin* **sp. nov.**; **Location:** country: China; stateProvince: Jiangxi; locality: Ganzhou City, Ruijin County Level City, Jiubao Town, Tongbo Mountain; verbatimElevation: 834 m; verbatimCoordinates: 26°01'26.79"N，115°50'19.91"E; georeferenceProtocol: GPS; **Event:** samplingProtocol: sieving; eventDate: 2/8/2025
- **Type status:** Paratype. **Occurrence:** catalogNumber: Phu-173; recordedBy: Liu Ke-Ke; individualCount: 1; sex: male; lifeStage: adult; occurrenceID: CB64FA93-B0C3-5EC0-9234-D23C35EA2B33; **Taxon:** scientificName: *Aculithusruijin* **sp. nov.**; **Location:** country: China; stateProvince: Jiangxi; locality: Ganzhou City, Ruijin County Level City, Jiubao Town, Tongbo Mountain; verbatimElevation: 834 m; verbatimCoordinates: 26°01'26.79"N，115°50'19.91"E; georeferenceProtocol: GPS; **Event:** samplingProtocol: sieving; eventDate: 2/28/2025
- **Type status:** Paratype. **Occurrence:** catalogNumber: Phu-173; recordedBy: Liu Ke-Ke; individualCount: 9; sex: female; lifeStage: adult; occurrenceID: 9F4D428B-28F2-535B-9D0B-30D2CC552BE1; **Taxon:** scientificName: *Aculithusruijin* **sp. nov.**; **Location:** country: China; stateProvince: Jiangxi; locality: Ganzhou City, Ruijin County Level City, Jiubao Town, Tongbo Mountain; verbatimElevation: 834 m; verbatimCoordinates: 26°01'26.79"N，115°50'19.91"E; georeferenceProtocol: GPS; **Event:** samplingProtocol: sieving; eventDate: 2/28/2025
- **Type status:** Paratype. **Occurrence:** catalogNumber: Phu-173; recordedBy: Liu Ke-Ke; individualCount: 6; sex: male; lifeStage: adult; occurrenceID: 225A01E8-9B62-56A1-A1E7-D24BC5830AF1; **Taxon:** scientificName: *Aculithusruijin* **sp. nov.**; **Location:** country: China; stateProvince: Jiangxi; locality: Ganzhou City, Ruijin County Level City, Jiubao Town, Tongbo Mountain; verbatimElevation: 833 m; verbatimCoordinates: 26°01'25.50"N，115°50'12.84"E; georeferenceProtocol: GPS; **Event:** samplingProtocol: sieving; eventDate: 2/28/2025
- **Type status:** Paratype. **Occurrence:** catalogNumber: Phu-173; recordedBy: Liu Ke-Ke; individualCount: 7; sex: female; lifeStage: adult; occurrenceID: E685797D-E166-50E5-BE6A-752E4069B3AA; **Taxon:** scientificName: *Aculithusruijin* **sp. nov.**; **Location:** country: China; stateProvince: Jiangxi; locality: Ganzhou City, Ruijin County Level City, Jiubao Town, Tongbo Mountain; verbatimElevation: 833 m; verbatimCoordinates: 26°01'25.50"N，115°50'12.84"E; georeferenceProtocol: GPS; **Event:** samplingProtocol: sieving; eventDate: 2/28/2025

#### Description

**Figure 3.**
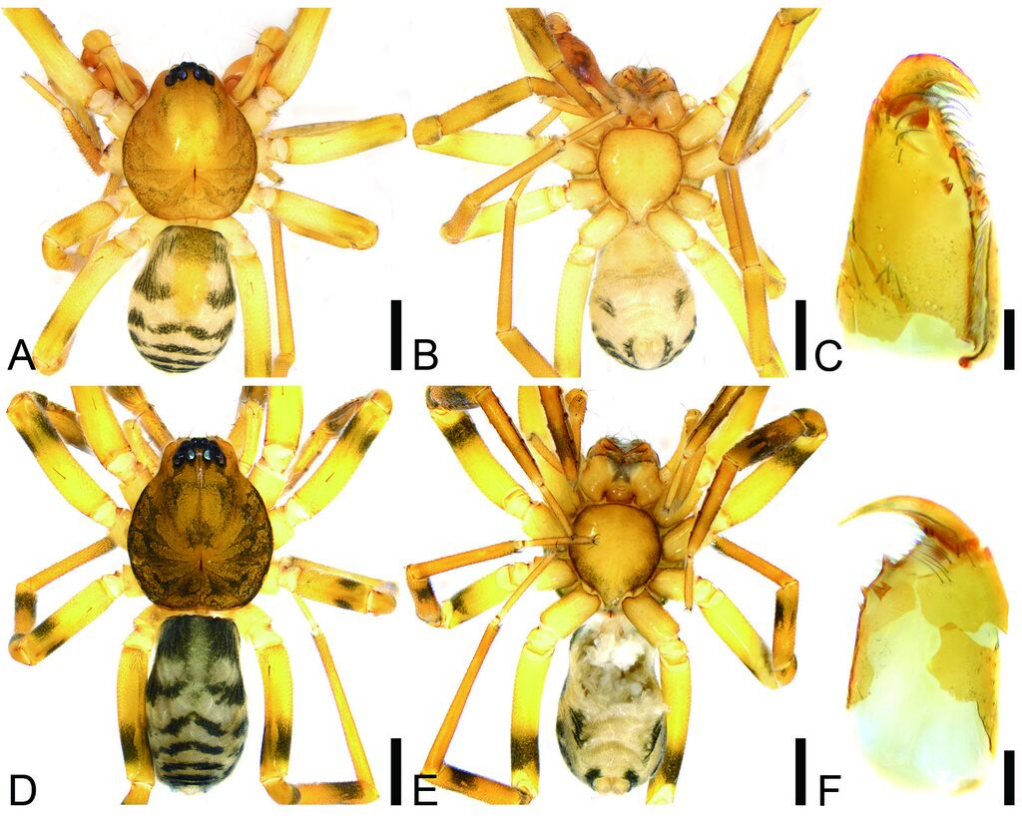
Habitus and chelicerae of *Aculithusruijin* **sp. nov.**. **A** Male (holotype), dorsal view; **B** Same, ventral view; **C** Right chelicera of male, ventro-retrolateral view; **D** Female, dorsal view; **E** Same, ventral view; **F** Left chelicera of female, ventro-retrolateral view. Scale bars: 0.5 mm (A−D), 0.1 mm (C−F).

**Male** (Holotype). Habitus as in Fig. 3A and B and Fig. 6E. Total length 2.28, carapace 1.17 long, 0.99 wide.

Eye sizes and interdistances Fig. 3A: AME 0.04, ALE 0.06, PME 0.06, PLE 0.07; AME−AME 0.03, AME−ALE 0.02, PME−PME 0.07, PME−PLE 0.05, AME−PME 0.07, AME−PLE 0.11, ALE−ALE 0.13, PLE−PLE 0.27, ALE−PLE 0.06. MOA 0.17 long, frontal width 0.1, posterior width 0.18. Chelicerae (Fig. 3C) with three promarginal (proximal largest, distal smallest and separate) and two retromarginal teeth (distal larger, compact). Sternum (Fig. 3B) slightly longer than wide, posteriorly triangular, relatively blunt. Leg measurements: I 3.68 (1.06, 0.32, 1.15, 0.72, 0.43); II 3.38 (0.87, 0.43, 0.83, 0.85, 0.4); III 2.9 (0.77, 0.35, 0.62, 0.77, 0.39); IV 4.31 (1.14, 0.4, 0.93, 1.2, 0.64). Left leg spination (Fig. 3A and B): femora I d11, p111, II d1, IV d1; tibiae I v222222, II v222222; metatarsi I v2222, II v2221. Pedicel 0.08 long. Abdomen (Fig. 3A and B) 1.03 long, 0.79 wide, dorsal scutum covering more than 1/2 length of abdomen.

Colouration (Fig. 3A and B). Carapace yellow, with irregular radial pale brown mottled markings around submargin and arc-shaped pale brown stripes along the margin. Chelicerae, endites and labium yellow. Sternum yellow, with yellow brown lateral margins. Legs yellow, with darker tibiae and metatarsi, distally bearing faint brown annulations. Abdomen yellowish-white, with a pair of large arc-shaped markings on both sides of the medial dorsal scutum, five dark brown wavy transverse stripes posteriorly and brown markings on the anterior half of the scutum; venter with a pair of slanting spots posterolaterally.

**Figure 4.**
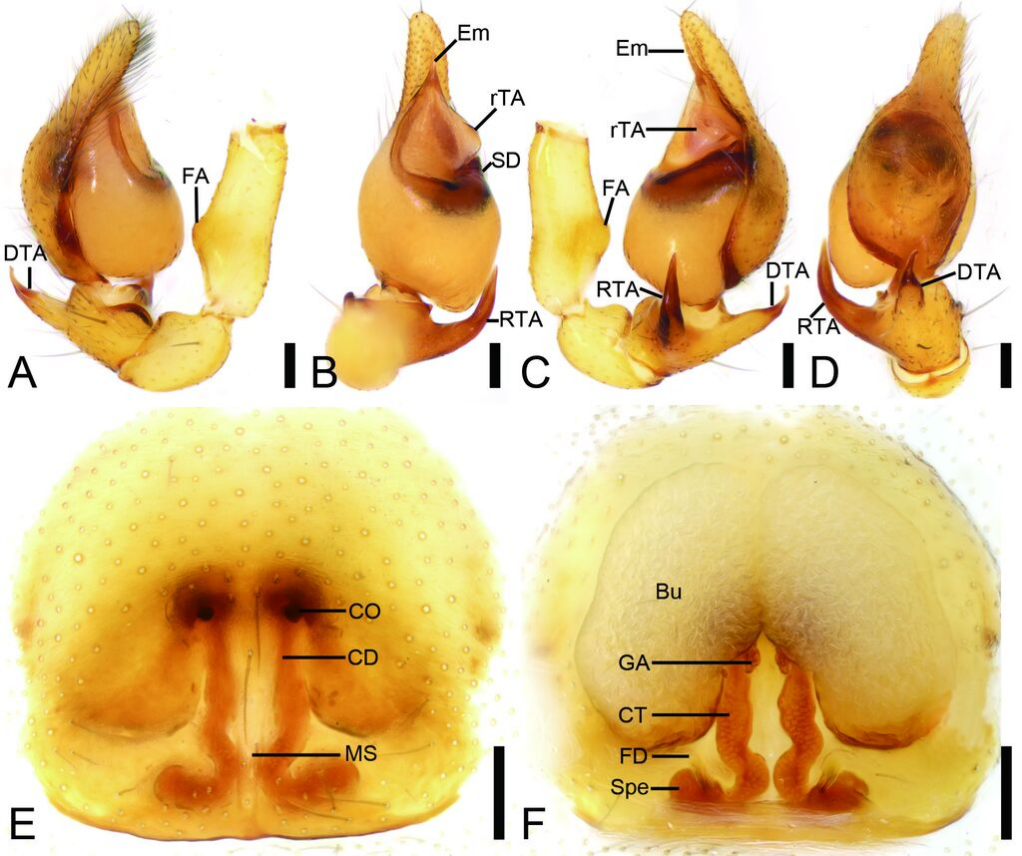
*Aculithusruijin* **sp. nov.**, male palp (holotype) and epigyne (paratype). **A** Left palp, prolateral view; **B** Same, ventral view; **C** Same, retrolateral view; **D** Same, dorsal view; **E** Epigyne, ventral view; **F** Vulva, dorsal view. Abbreviations: Bu − bursa, CD − copulatory duct, CO − copulatory opening, CT − connecting tube, DTA − dorsal tibial apophysis, Em − embolus, FA − femoral apophysis, FD − fertilisation ducts, GA − glandular appendage, MS − median septum, rTA − retrolateral tegular apophysis, RTA − retrolateral tibial apophysis, Spe − spermathecae. Scale bars: 0.1 mm (A−F).

**Figure 5.**
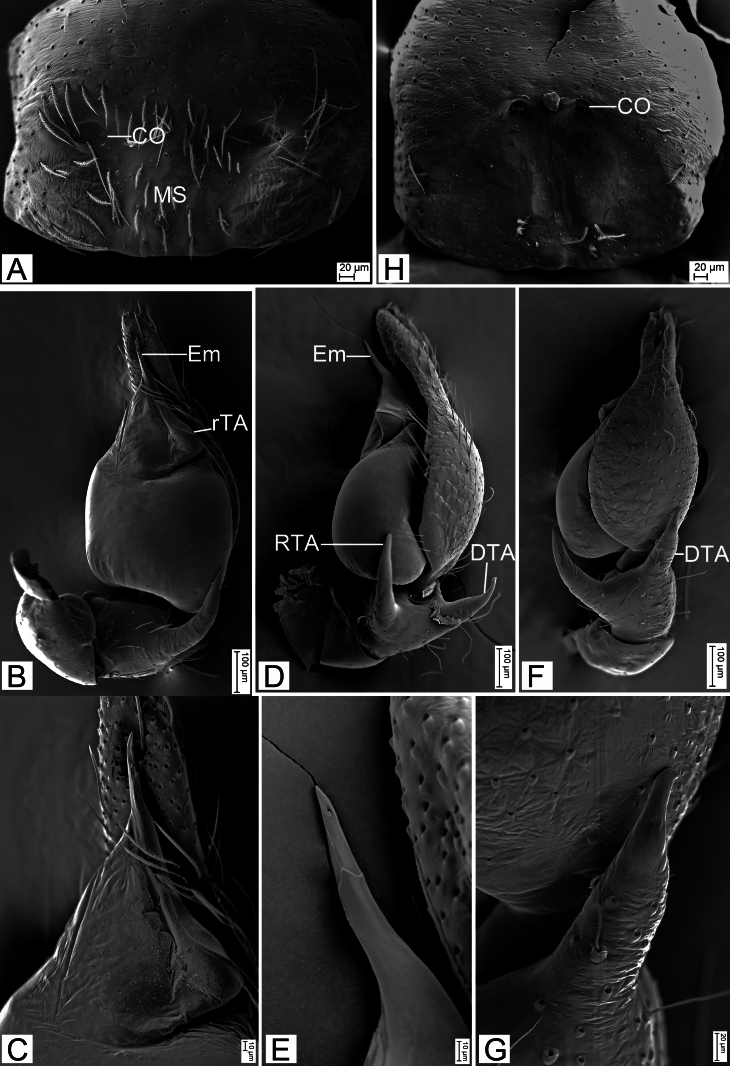
*Aculithuslanguan* **sp.nov.** and *A.ruijin* **sp. nov.**, paratypes. **A** *A.languan* **sp.nov.**, epigyne, ventral view; **B** *A.ruijin* **sp. nov.**, left palp, ventral view; **C** Same, detail of Em and rTA, ventral view; **D** Same, retrolateral view; **E** Same, detail of Em, retrolateral view; **F** Same, dorsal view; **G** Same, detail of DTA, dorsal view; **H** Same, epigyne, ventral view. Abbreviations: CO − copulatory opening, DTA - dorsal tibial apophysis, Em - embolus, MS − median septum, rTA −retrolateral tegular apophysis, RTA − retrolateral tibial apophysis.

Palp (Fig. 4A−D and Fig. 5B−G). Femoral apophysis (FA) strong, not sclerotised, width less than half of its length. Retrolateral tibial apophysis (RTA) large, finger-shaped, longer than tibia, strongly sclerotised, apex pointed, bent ventrally. Dorsal tibial apophysis (DTA) shorter than retrolateral tibial apophysis, curved prolaterally, sclerotised and tapered at the subapex. Sperm duct (SD) nearly C-shaped, extending from below the retrolateral tegular apophysis (rTA) and gradually narrowing. Retrolateral tegular apophysis relatively broad, membranous, protruding and blunt retrolaterally. Embolus (Em) relatively long, spine-like.

**Figure 6.**
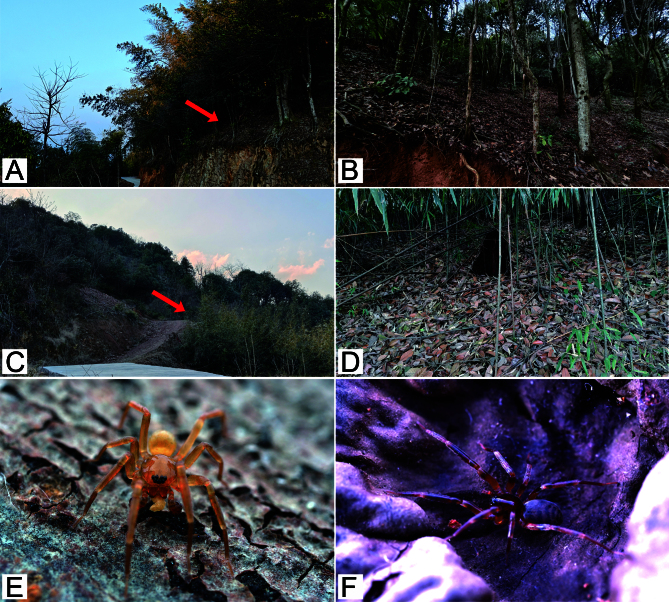
Habitat and live specimens of *Aculithusruijin* **sp. nov.**. **A**−**D** Habitat, red arrows show the sampling location; **E** Male; **F** Female.

**Female** (Paratype). Habitus as in Fig. 3D and E and Fig. 6F. Total length 2.7, carapace 1.29 long, 1.12 wide. Eye sizes and interdistances: AME 0.05, ALE 0.07, PME 0.07, PLE 0.09, AME-AME 0.02, AME-ALE 0.03, PME-PME 0.06, PME-PLE 0.04, AME-PME 0.06, AME-PLE 0.14, ALE-ALE 0.14, PLE-PLE 0.28, ALE-PLE 0.06. MOA 0.17 long, frontal width 0.11, posterior width 0.21. Leg measurements: I 4.46 (1.16, 0.5, 1.28, 1.09, 0.43); II 3.77 (0.98, 0.47, 0.95, 0.9, 0.47); III 3.44 (0.85, 0.42, 0.7, 0.89, 0.58); IV 4.7 (1.26, 0.43, 1.03, 1.34, 0.64). Left leg spination (Fig. 3D, E): femora I d11 p111, II d1, III d1, IV d1; tibiae I v22222221, II v2222221, metatarsi I v2222, II v2221. Pedicel 0.06 long. Abdomen (Fig. 3D and E) 1.35 long, 0.85 wide.

Colouration (Fig. 3D and E). Darker than male, with legs I-IV bearing black annulations distally on femora, patellae and tibiae, while metatarsi I-II with blackish-brown mottled markings distally.

Epigyne (Fig. 4E and F and Fig. 5H). Epigynal plate mushroom-shaped, antero-medially with pair of small, oval copulatory openings (CO), medially with indistinct median septum (MS). Copulatory ducts (CD), connecting tubes (CT) and spermathecae (Spe) distinctly visible through integument in intact epigyne. Copulatory ducts shorter than half length of connecting tubes, located between copulatory openings and glandular appendages, both sides with pair of large, bean-shaped, transparent bursae (Bu), covering more than half of the epigyne. Glandular appendages (GA) longer than half the width of connecting tubes, near base of bursae, located on anterior of connecting tubes. Connecting tubes approximately half length of the bursae, with the anterior part nearly parallel and the posterior part S-shaped. Spermathecae oval, directed laterally. Fertilisation ducts (FD) as long as spermathecae, directed anteriorly and located near the posterior end of the vulva.

#### Diagnosis

The males of the new species are similar to *A.subfabiformis* (Liu, 2020) in having a spine-like embolus, a C-shaped sperm duct and a broad retrolateral tegular apophysis (see Liu et al. (2020): 14, fig. 9), but can be separated from it by the retrolateral tegular apophysis with a broad retrolateral part (vs. thin), the dorsal tibial apophysis sharp narrowing medially (vs. gradually tapered) and the slender embolus (vs. short embolus) (cf. Fig. 4B−C and Fig. 5B−F and Liu et al. (2020): 14, fig. 9D). The female resembles *A.subfabiformis* (Liu, 2020) in having the large bursae covering more than half of the epigyne and the slightly curved and medially separated connecting tubes (see Liu et al. (2020): 16, fig. 11), but can be separated from it by the bursae separated posteriorly (vs. compact posteriorly) and the wide separated spermathecae (vs. closely touching) (cf. Fig. 4E and Liu et al. (2020): 14, fig. 11D).

#### Etymology

The species name is derived from the name of the type locality; noun in apposition.

#### Distribution

**Figure 7.**
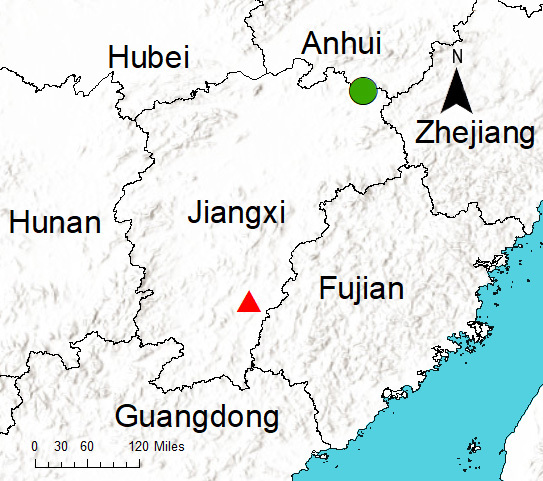
Records of *Aculithuslanguan* **sp. nov.** (green circle) and *A.ruijin* **sp. nov.** (red triangle) from Jiangxi Province, China.

Known only from the type locality in Jiangxi Province, China (Fig. 7).

#### Biology

It was collected from leaf litter in areas of broad-leaved forests and bamboo broadleaf mixed forests in hilly areas (Fig. 6A−D).

### Aculithus taoyuan

(Fu, Chen & Zhang, 2016) comb. nov.

EAB42D1B-99B5-57B0-9169-A7D95BC3EEC9

*Phrurolithustaoyuan* Fu, Chen & Zhang, 2016 Fu et al. (2016): 283, figs. 9A−H, 10A−E (♂♀, type deposition in MHBU).
*Otaciliataoyuan* (Fu, Chen & Zhang, 2016) Zamani and Marusik (2020): 312.

#### Description

See *Fu et al. (2016)*.

#### Diagnosis

Male of this species (see Fu et al. (2016): 283, figs. 9A−H and 10A−E) similar to other species of *Aculithus* in having irregular radial dark stripes mediolaterally on carapace, arc-shaped dark stripes along margin, small PME, large retrolateral and dorsal tibial apophyses, short spine-like embolus and membranous retrolateral tegular apophysis, but can be distinguished by retrolateral and dorsal tibial apophyses with sudden mid-length constriction (vs. gradually tapered), elongated retrolateral tegular apophysis (vs. short and thick). For females, see the diagnosis of *A.languan* **sp. nov.** (cf. Fu et al. (2016): 283, fig. 9H and Fig. 1E).

#### Distribution

Known only from the type locality in Sichuan Province, China.

## Supplementary Material

XML Treatment for Aculithus languan

XML Treatment for Aculithus ruijin

XML Treatment for Aculithus taoyuan
